# Association of Endothelin-Converting Enzyme and Endothelin-1 Gene Polymorphisms with Essential Hypertension in Malay Ethnics

**DOI:** 10.1155/2022/9129960

**Published:** 2022-05-16

**Authors:** Elnaz Salim, Vasudevan Ramachandran, Neda Ansari, Patimah Ismail, Mohd Hazmi Mohamed, Nur Afiqah Mohamad, Liyana Najwa Inche Mat

**Affiliations:** ^1^Department of Biomedical Science, Faculty of Medicine and Health Sciences, Universiti Putra Malaysia, Serdang 43400, Selangor, Malaysia; ^2^Centre for Materials Engineering and Regenerative Medicine, Bharath Institute of Higher Education and Research, Selaiyur, Chennai 600073, Tamil Nadu, India; ^3^Department of Surgery, Faculty of Medicine and Health Sciences, Universiti Putra Malaysia, Serdang 43400, Selangor DE, Malaysia; ^4^Department of Neurology, Faculty of Medicine and Health Sciences, Universiti Putra Malaysia, Serdang 43400, Selangor DE, Malaysia; ^5^Center of Foundation Studies, Lincoln University College, Petaling Jaya 47301, Selangor DE, Malaysia; ^6^Department of Medicine, Faculty of Medicine and Health Sciences, Universiti Putra Malaysia, Serdang 43400, Selangor DE, Malaysia

## Abstract

**Objectives:**

Endothelin-1 (ET-1), the most potent endogenous vasoconstrictor, generated by enzymatic cleavage catalyzed by an endothelin-converting enzyme (ECE), plays a significant role in the regulation of hypertension.

**Methods:**

This study investigates the effect of endothelin-1 (Lys198Asn/rs5370) and ECE (rs212526 C/T) gene polymorphisms with essential hypertension (EH) among Malay ethnics. To determine the association of gene polymorphism, 177 hypertensives and controls (196) were genotyped using *Taqman* method.

**Results:**

A significant difference was observed in ET-1 rs5370 and ECE rs212526 gene polymorphisms between EH and control subjects (*P* < 0.001). A significantly high body mass index (BMI), waist-to-hip ratio, fasting plasma glucose, hemoglobin A1c, systolic and diastolic blood pressure, and lipid profiles were observed among the EH patients when compared to controls (*P* < 0.05). Moreover, T allele (rs5370) carriers in males have a high risk for EH. There was no significant association between gender in ECE C/T polymorphisms (*P* > 0.05).

**Conclusion:**

Based on our result, it is evident that the T allele of ET-1 rs5370 polymorphism and C allele of ECE rs212526 have a significant genetic risk factor in EH among Malay subjects, and BMI and age are associated with hypertension.

## 1. Introduction

Sociodemographic factors are highly contributing to the development of essential hypertension (EH) and have been documented in several epidemiological studies, making it a complex disease via interaction between genetic and environmental risk factors [1]. The EH is a multifaceted disease with an interaction between genetic and environmental risk factors [2]. Patients with EH typically have no symptoms; however, they could experience frequent headaches, exhaustion, dizziness, or bleeding of the nose [3]. Hypertension is the most important risk factor for the development of cardiovascular disease, stroke, and renal disease [4, 5]. Apart from that, the essential risk factors of EH are positive family history, obesity, and physical inactivity [6]. Several research studies have initiated the identification of genetic variants across the entire genome predisposing to EH. Researchers have identified several EH risk loci using various technologies in various populations [7]. However, the knowledge of the specific causes of common complex diseases at the genetic level remains unclear. Identifying the genetic risk factors and underlying molecular mechanisms associated with EH may allow further therapeutic clinical intervention and investigations in preventing the disease. This makes it a vital challenge for researchers to identify the susceptibility genes associated with EH to elucidate the complex pathogenesis of EH [7, 8]. Endothelin is a chemical formed by the endothelin cells and originally disengaged from the supernatant of porcine aortic endothelial culture and showed strong vasoconstrictive peptide [9, 10]. Endothelin has 3 isoforms (ET-1, ET-2, and ET-3) and 21 amino acid peptides translated from 3 autonomous genes and distributed by endothelium in various tissues [11]. Endothelin-1 (ET-1) is the most active and predominant endothelin isoform in the human cardiovascular system. It is now recognized as playing a pivotal role in the vascular tone control, cell proliferation, acid and base handling, and regional blood flow [12]. Moreover, the ET-1 gene also increased the pathophysiology of several diseases including ischemic heart, arteriosclerosis, hypertension, and diabetes [13]. The concentration of ET-1 in the blood increases by standing and decreases by volume overload and is considered modulated by the amount of body fluid via the renin-angiotensin-aldosterone system [14]. The ECE-1 is an isoform and membrane-bound metalloprotease expressed in various cells and tissues [15, 16], while ECE-2 is an intracellular processing enzyme that works under acidic conditions and is primarily expressed in neurons. The ECE gene contributes to the generation of vascular ET and blood pressure (BP) regulation regulated by variation in its constitutive expression level or mechanisms dependent on the protein kinase C [17]. Therefore, ECE and ET-1 genes have been proposed as a candidate's risk factor for hypertension. Gene polymorphism (Lys198Asn) of ET-1 gene is correlated with EH in overweight and elderly subjects [13, 18]. Single-nucleotide variants could affect the ECE1 mRNA level, then affect the function of this enzyme, and eventually induce changes in the ET expression level [19]. The rs212526 C/T and rs5370 polymorphisms of ECE-1 gene are associated with BP regulation [20]. Studies made significant contributions because they provided us with a hint that endothelin gene SNP may be correlated with EH or any other diseases linked to BP [21]. However, a few studies have reported the association with conflicting results in various populations, and it might be due to the ethnic background and environmental factors [13, 18, 20, 21]. Hence, ET-1 rs5370 and ECE-1 rs212526 gene polymorphisms' susceptibility to hypertension among Malay ethnics is analyzed in this study.

## 2. Methods

### 2.1. Study Population

Upon acceptance from Ethics Committee, Ministry of Health Malaysia [NMRR-16-240-29375], a total number of 373 individuals, including 177 case subjects with hypertension and 196 control subjects without hypertension, is recruited from Hospital Serdang Malaysia. Sociodemographic and medical records were recorded. Newly diagnosed hypertensive patients with systolic blood pressure (SBP) of 140 mmHg and/or diastolic blood pressure (DBP) more than 90 mmHg on two or more consecutive visits were recruited as case subjects. Patients with a history of acute myocardial infarction, renal failure, cardiac failure or diabetes, pregnancy, and lactation and patients under medications for other indications that could affect BP were excluded. Healthy volunteers with no medical or family history of hypertension were recruited as controls.

### 2.2. DNA Extraction

Genomic DNA was extracted from the blood using the DNA extraction kit (QIAGEN) according to the manufacturer's protocol. The isolated DNA was quantified with a Nano-Drop 1000 spectrophotometer (Thermo Scientific, USA).

### 2.3. Genotypic Analysis

Endothelin-1 and ECE genes were amplified by a *Taqman* probe (PE Applied Biosystems) using real-time polymerase chain reaction (qPCR). A fluorescent reporter dye, 6-carboxy-fluorescein (FAM), was linked covalently to the 5 ends of the nucleotide. Probes for ET1 gene polymorphism (forward primer-rhAmp-F/ATCCCAAGCTGAAAGGCAAGCCCTC/GT3, reverse primer-rhAmp-Y/ATCCCAAGCTGAAAGGCAATCCTC/GT3) and ECE gene polymorphism (forward primer-rhAmp-F/GCCCAGGAGTTGACATCATUCTTG/GT2, rhAmp-Y/GCCCAGGAGTT GACATCATUCTTG/GT2) were designed.

DNA samples were amplified in a 5 *μ*l reaction consisting of 2.65 *μ*l combined mix (reporter mix and master mix), 0.25 *μ*l rhAmp primer as well as 2.10 *μ*l nuclease-free water. Real-time PCR was executed in triplicate for each DNA sample with primer corresponding to the gene of interest. A nontemplate control (NTC) using PCR-grade water was included. The PCR cycling parameters were as follows: an initial holding step for enzyme activation for 10 min at 95°C and 35 cycles denaturation step 10 sec at 95°C and annealing 30 sec at 60°C followed by the extension step 20 sec at 68°C final holding for inactivation of enzyme 15 mins at 99°C.

### 2.4. Statistical Analysis

Statistical analyses were performed using Statistical Package for the Social Sciences (SPSS) version 21.0. Continuous variables were presented as mean ± standard deviation, and categorical variables were presented as percentages. Students' *t*-test was used to test means between groups. The Hardy–Weinberg equilibrium of alleles in each SNPs was confirmed using the Haploview software (https://www.broad.mit.edu/mpg/haploview), and allelic frequencies were calculated by gene counting method. The chi-square test was applied for the categorical variables. ANOVA test was used to analyze significant differences between means and to indicate the difference in clinical and biochemical outcomes of individuals compared with genotypes. Multiple binary logistic regression was employed to evaluate the effect of genotype and allele on the outcome. A *P* value of <0.05 was considered statistically significant.

### 2.5. Ethics Approval

Ethical approval has been obtained from the Ministry of Health Medical Research and Ethical Committee (MREC) Board Reference number (NMRR-16-240-29375). Written informed consent was obtained from all the participants.

## 3. Results

### 3.1. Characteristics of the Study Subject

A total of 373 samples were included for genotyping of ET-1 rs5370 and ECE rs212526. The subject's sociodemographic characteristics are presented in Table 1. The number of male (59.9%) subjects was higher than that of female (40.0%) subjects among the EH patients. The lipid profile values such as total cholesterol (TC), triglyceride (TG), high-density lipoprotein cholesterol (HDL), and low-density lipoprotein cholesterol (LDL) were comparable in patients and controls (*P* < 0.001). The SBP in patients was significantly higher (156.80 ± 12.13) than that of controls (121.52 ± 10.24, *P* < 0.001). Similarly, DBP in patients was higher (94.49 ± 5.8, *P* < 0.001). Body mass index (BMI), fasting plasma glucose (FPG), hemoglobin A1c (HbA1c), waist-to-hip ratio (WHR), TG, and cholesterol are highly significant (*P* < 0.05) between EH and control subjects.

### 3.2. Distribution of Genotype and Allele Frequencies

The *Taqman* qPCR method demonstrates the genotype distribution of rs5370 polymorphism of the ET-1 and ECE C/T rs212526 genes between EH and control subjects. The deviation from H-W equilibrium for both gene ET1 (*χ*^2^ = 134.071, *P* < 0.001) and ECE (*χ*^2^ = 525.772, *P* < 0.001) was statistically significant. There are three possible genotypes, including TT (mutant, HOM MT), GT (heterozygote, HET), and wild-type GG (wild type, WT). A statistically significant difference was observed in genotype frequencies between the hypertensive and healthy subjects (*P* < 0.001). Based on Table 2, the frequencies of TT, GT, and GG are 65.0%, 26.0%, and 9.0%, respectively, in the case group and 8.7%, 15.8%, and 75.5%, respectively, in the control group. Results of binary logistic regression indicated that compared to GG (as the reference group), the odd ratio of genotype TT significantly increased the chance of hypertensive status (OR = 62.574, 95% CI = 30.31–129.179, *P* < 0.001). According to these results, the chance of hypertensive status in heterozygous genotype (GT) compared to GG increased significantly (OR = 13.726, 95% CI = 6.898–27.313, *P* < 0.001). The allele frequency of ET-1 rs5370 was associated with hypertensive status (OR = 17.801, 95% CI = 12.344–25.671, *P* < 0.001). The T (78%) allele frequency in the EH group was higher than that in the normotensive group (16.6%). In the dominant and recessive models, the genotype frequencies TT + GT and GG + GT were 91.00% and 35.0%, respectively, among the case subjects and 24.5% and 91.3%, respectively, in controls (*P* value = 0.001). Overall, TT genotype was associated with an increased risk of EH. A post hoc test was conducted between EH and control subjects that indicated significance between WT and HET (*P* value = 0.001), followed by WT and HOM MT (*P* value = 0.001), as shown in Table 3.

Besides, three genotypes were presented for the ECE rs212526 gene polymorphism: CC (mutation, HOM MT), CT (heterozygous, HET), and TT (wild type, WT). Based on the results presented in Table 4, the frequency of heterozygotes genotype CT (23.2%) was higher than the cases rather than in the healthy controls (8.2%). Results of binary logistic regression indicated that compared to TT (as the reference group), the presence of genotype CT significantly increased the chance of hypertensive status (OR = 3.679, 95% CI = 1.971–6.868, *P* < 0.001). This result indicated that mutation CC (10.7%) EH was higher than in the normotensive group (6.1%), and wild-type TT in the control group (85.7%) was higher than cases (66.1%). In this study, the allele frequencies of ECE rs212526 polymorphism showed that carrier with C allele (23.3.%) was significantly associated with EH (OR = 2.528, 95% CI = 1.675–3.816, P < .001), as it is higher than the controls (10.2%). As shown in Table 4, the genotype frequency of ECE C/T rs212526 was associated with EH in dominant and recessive (*P*=0.001) models. The post hoc test was also conducted between EH and control subjects that indicated significance between WT and HET (*P* value = 0.001), followed by WT and HOM MT (*P* value = 0.001).

An ANOVA test was performed using the general linear model to find the confounding factors for ET-1 rs5370 and ECE rs212526 gene polymorphism (Table 4). Significant differences (*P* < 0.05) were found between genotypes in BMI, SBP, DSB, LDL, cholesterol, and HbA1c among control subjects, and BMI, WHR, FPG, and LDL among cases according to ET-1 rs5370 genotypes. However, the clinical parameters between ECE rs212528 genotypes showed a significant difference for age, WHR, and DBP among the controls. In contrast, cases showed a significance for BMI only, with the mutant C allele associated with high BMI (Table 5).

We further examined our data and divided the total group by gender (age and BMI as covariates) and analyzed ET-1 rs5370 and ECE rs212526 genotype frequencies for both genders separately. As shown in Table 6, in the male group, TT genotype compared with GG genotype (wild type) was higher than in the female. However, no significant difference was found between genders. In addition, GT genotype in the male also was a higher chance of hypertension when compared to GG as the reference group (OR = 23.178, 95% CI = 5.618–95.629, *P* < 0.001). However, there was no significant difference in GT genotype in the female groups by *P* > 0.05. This suggested that male subjects with T allele carriers of rs5370 have high risk for EH. However, the genotype frequencies of ECE C/T rs212526 are significantly different between males and females by *P* > 0.05, compared with combined group of CT with TT wild-type carriers (men; OR = 1.306, 95% CI = 0.103–16.574, female OR = 0.751, 95% CI = 0.083–6.809) or CC with wild-type TT (men: OR = 0.83, 95% CI = 0.078–8.847, female” OR = 0.348, 95% CI = 0.05–2.404).

## 4. Discussion

Association studies are very important for a better understanding about the interaction between genes and diseases, despite the abundance of animal and human models, and pharmacological interventions studies support the role of the endothelin genes as a major factor in hypertension, and the direct interaction of ET-1 plasma levels to hypertension remains controversial [5, 21].

In this study, the variants of ET-1 (rs5370) and ECE (rs212526) genes in Malay ethnics were analyzed. The allelic and genotypic frequencies were analyzed between the cases (177) and control subjects (196). Significant differences were noticed in both gene polymorphisms (*P* < 0.05), with the mutant C allele of ECE rs212526 and T allele of ET-1 rs5370. These results suggest that these two polymorphisms play a major role in developing hypertension in Malay ethnics.

Several studies have described the ET-1 rs5370 polymorphism as positively correlated with BP and BMI, such as the Caucasians and Japanese [21]. Hence, ET-1 rs5370 polymorphism can be considered as a possible risk factor, with T allele being associated with high BP in several studies such as European, Australian, Japanese, and American populations [22]. The Asn allele of ET-1 gene was associated with hypertension and homozygotes Asn 198 with a significantly evaluated plasma level of ET-1 [23]. A report suggested ET-1's vasoconstrictive effect in male rats to be as much greater than female rats [24]. A study found that male hormones could increase plasma ET expression levels while estrogen would lower the plasma ET levels and could alter ET's regulatory activity on renal water and salt metabolism [25]. Our current findings indicate that rs5370 polymorphism affected BP regulation through the production of ET-1 and significant differences between gender. However, the functional consequence of the rs5370 polymorphism remains unknown.

ET-1 is generated by cleavage of the inactive precursor preproendothelin-1 (ppET-1) by a furin-like endopeptidase to form the intermediate pET-1 that is finally cleaved by the specific metalloendopeptidase ECE-1 to yield active ET-1 [26]. Thus, ECE-1 is an important regulator of ET-1 generation and thereby of BP [27]. This polymorphism is associated with higher BP levels in patients, most probably due to an increased promoter activity [28].

The physiological importance of ET is not yet completely comprehended. Despite its vasoconstrictive properties, ET additionally stimulates the arrangement of nitric oxide along these lines restricting vasoconstriction [29]. In any case, the expanded plasma levels of ET are seen in patients with optional types of hypertension, including renal and pulmonary hypertension. In the perspective of the generous effect of ET on vascular tone and endothelial gene function, variations of the ET and ECE gene might be causally identified with the improvement and movement of major cardiovascular diseases. A few limitations must be considered in this study. Firstly, there is a significant difference in the age between groups since the majority of Malay patients at Hospital Serdang are hypertensive and elderly. Secondly, two common gene polymorphisms of ET-1 and ECE gene polymorphisms were only analyzed with EH among Malay ethnic subjects. Analyzing various gene polymorphisms of candidate genes with a larger number of samples is strongly recommended.

## 5. Conclusion

There is a significant association between ET-1 rs5370 and ECE rs212526 gene polymorphisms with hypertension in the Malay ethnic. Future studies with a larger sample size are recommended to examine the variants of ET-1 and ECE towards the development of hypertension.

## Figures and Tables

**Table 1:** Clinical and biochemical parameter of EH patients and control subjects.

Parameter	Control (%)	Case (%)	*P*
No. of samples	196	177	
Age	37.17 ± 9.408	57.02 ± 9.516	<0.001
Gender			
Male	98 (50.0%)	106 (59.9%)	0.056
Female	98 (50.6%)	71 (40.0%)	
BMI	23.58 ± 3.82	28.77 ± 4.64	<0.001
WHR	0.91 ± 0.14	0.899 ± 0.098	<0.001
FPG	5.50 ± 1.38	6.55 ± 2.42	<0.001
HbA1c	2.73 ± 2.93	6.47 ± 1.87	<0.001
SBP	121.52 ± 10.24	156.80 ± 12.13	<0.001
DBP	78.21 ± 7.43	94.49 ± 5.8	<0.001
Cholesterol	4.83 ± 0.93	5.04 ± 1.10	0.049
LDL	2.63 ± 0.94	2.86 ± 0.88	0.20
HDL	1.10 ± 0.35	1.25 ± 0.74	0.015
TG	1.46 ± 0.65	1.85 ± 0.99	<0.001

Note: BMI = body mass index; WHR = waist-to-hip ratio; FPG = fasting plasma glucose; HbA1c = hemoglobin A1c; SBP = systolic blood pressure; DBP = diastolic blood pressure; Chol = cholesterol; LDL = low-density lipid; HDL = high-density lipid; TG = triglyceride.

**Table 2:** Genotype and allele frequencies of ET-1 rs5370 polymorphism.

Group		Control (%)	Case (%)	*P* ^a^	OR	95% CI LL	95% CI UL
Genotype	TT	17 (8.7)	115 (65.0)	<0.001	62.574	30.31	129.179
	GT	31 (15.8)	46 (26.0)		13.726	6.898	27.313
	GG	148 (75.5)	16 (9.0)		Ref		

Allele							
	G	327 (83.4)	78 (22)	<0.001	Ref 17.801	12.344	25.671
	T	65 (16.6)	276 (78)				
DOM	TT + GT	48 (24.5)	161 (91.00)	<0.001			

RES	GG + GT	179 (91.3)	62 (35.0)	<0.001			
	Post hoc test	Χ^2^	*P* value^a^				
	GG vs. GT	30.8	0.00001				
	GG vs. TT	120.65	0.00001				
	GT vs. TT	31.672	0.00001				

Note: HOM MT = Homozygous mutant; HET = heterozygous; WT = wild type; DOM = dominant; RES = recessive. ^a^Chi-square test.

**Table 3:** Genotype and allele frequencies of ECE rs212526 polymorphism.

Group		Control (%)	Case (%)	*P* ^a^	OR	95% CI LL	95% CI UL
Genotype	CC	12 (6.1)	19 (10.7)	<0.001	2.274	1.063	4.863
	CT	16 (8.2)	41 (23.2)		3.679	1.971	6.868
	TT	168 (85.7)	117 (66.1)		Ref		

Allele							
	C	40 (10.2)	79 (23.3)	<0.001	2.528	1.675	3.816
	T	352 (89.8)	275 (77.7)		Ref		
DOM	CC + CT	28 (14.3)	60 (33.9)	<0.001			

RES	TT + CT	184 (93.9)	158 (89.3)	<0.001			
	Post hoc test	Χ^2^	*P* value^a^				
	CC vs. CT	0.811	0.36				
	CC vs. TT	8.6	0.0033				
	CT vs. TT	10.156	0.0014				

Note: HOM MT = Homozygous mutant; HET = heterozygous; WT = wild type; DOM = dominant; RES = recessive. ^a^Chi-square test.

**Table 4:** Clinical and biochemical characteristics according to genotypes of ET-1 rs5370 gene polymorphism.

Parameter	Control (mean ± SD)				Case (mean ± SD)			
	TT	GT	GG	*P* ^a^	TT	GT	GG	*P* ^a^
Age	36.41 ± 9.36	39.06 ± 9.64	40.35 ± 8.74	0.124	55.25 ± 8.37	56.33 ± 10	57.55 ± 9.5	0.565
BMI	22.71 ± 2.77	26.9 ± 5.63	25.12 ± 4.22	<0.001	23.81 ± 1.78	28.03 ± 4.17	29.76 ± 4.62	<0.001
WHR	0.92 ± 0.15	0.9 ± 0.16	0.88 ± 0.06	0.583	0.83 ± 0.06	0.91 ± 0.06	0.9 ± 0.11	0.016
FPG	5.39 ± 1.21	5.96 ± 1.92	5.68 ± 1.65	0.117	5.33 ± 2.98	6.22 ± 1.6	6.87 ± 2.56	0.041
HBA1C	2.35 ± 2.78	4.91 ± 2.67	2.27 ± 3.23	<0.001	6.29 ± 1.57	6.02 ± 1.44	6.69 ± 2.04	0.115
SBP	119.98 ± 9.64	125.16 ± 9.57	128.29 ± 12.58	<0.001	153.19 ± 10.02	159.15 ± 12.77	156.37 ± 12.06	0.194
DBP	77.45 ± 7.42	80.19 ± 7.34	81.29 ± 6.66	0.035	93.88 ± 5.43	95.8 ± 6.09	94.05 ± 5.75	0.207
CHOL	4.88 ± 0.96	4.56 ± 0.87	4.95 ± 0.83	<0.001	4.65 ± 0.95	4.94 ± 1.14	5.14 ± 1.1	0.188
LDL	2.62 ± 1	2.61 ± 0.73	2.88 ± 0.86	<0.001	2.57 ± 0.53	2.65 ± 0.92	2.99 ± 0.89	0.033
HDL	1.1 ± 0.36	1.16 ± 0.32	1.02 ± 0.36	0.395	1.16 ± 0.31	1.3 ± 0.46	1.24 ± 0.87	0.809
TG	1.51 ± 0.66	1.27 ± 0.49	1.48 ± 0.82	0.173	1.97 ± 1.08	1.65 ± 0.84	1.92 ± 1.04	0.279

Note: SD = Standard deviation; BMI = body mass index; WHR = waist-to-hip ratio; FPG = fasting plasma glucose; HbA1c = hemoglobin A1c; SBP = systolic blood pressure; DBP = diastolic blood pressure; Chol = cholesterol; LDL = low-density lipid; HDL = High-density lipid; TG = triglyceride. ^a^One-way ANOVA.

**Table 5:** Clinical and biochemical characteristics according to genotypes of ECE rs212526 gene polymorphism.

Parameter	Control (mean ± SD)				Case (mean ± SD)			
	CC	CT	TT	*P* ^a^	CC	CT	TT	*P* ^a^
Age	33.58 ± 4.76	42.56 ± 11.4	36.92 ± 9.3	0.028	58 ± 4.68	57.32 ± 9.39	56.76 ± 10.16	0.85
BMI	22 ± 1.36	24.74 ± 4.64	23.59 ± 3.84	0.174	31.66 ± 4.41	28.75 ± 4.88	28.31 ± 4.47	0.014
WHR	0.86 ± 0.08	1.03 ± 0.27	0.9 ± 0.13	0.002	0.86 ± 0.23	0.91 ± 0.06	0.9 ± 0.06	0.24
FPG	5.19 ± 0.91	5.75 ± 1.16	5.51 ± 1.44	0.59	7.71 ± 3.4	6.65 ± 2.49	6.34 ± 2.19	0.104
HbA1c	1.04 ± 2.33	3.37 ± 3.11	2.79 ± 2.93	0.091	6.26 ± 3.2	6.92 ± 1.94	6.37 ± 1.53	0.248
SBP	114.67 ± 10.75	121.44 ± 10.6	122.02 ± 10.06	0.055	156.53 ± 9.77	157.88 ± 12.09	156.48 ± 12.56	0.814
DBP	72.58 ± 5.81	77.13 ± 7.24	78.73 ± 7.41	0.017	92.42 ± 5.95	95.95 ± 5.7	94.33 ± 5.78	0.082
Chol	5.14 ± 1.04	4.73 ± 1.32	4.82 ± 0.89	0.468	4.95 ± 1.02	5.11 ± 1.25	5.04 ± 1.07	0.871
LDL	3.15 ± 0.88	2.34 ± 0.91	2.63 ± 0.95	0.074	2.76 ± 0.83	2.75 ± 0.98	2.92 ± 0.86	0.492
HDL	1.16 ± 0.29	1.04 ± 0.41	1.1 ± 0.35	0.664	1.28 ± 0.4	1.19 ± 0.35	1.27 ± 0.88	0.854
TG	1.64 ± 0.53	1.4 ± 0.44	1.46 ± 0.68	0.621	1.68 ± 0.88	1.92 ± 1.22	1.86 ± 0.93	0.679

Note: SD = standard deviation; BMI = body mass index; WHR = waist-to-hip ratio; FPG = fasting plasma glucose; HbA1c = hemoglobin A1c; SBP = systolic blood pressure; DBP = Diastolic blood pressure; Chol = cholesterol; LDL = low-density lipid; HDL = high-density lipid; TG = triglyceride. ^a^One-way ANOVA.

**Table 6:** Genotypic distribution in female and male.

Gene	Gender	Genotype	Control *n* (%)	Case *n* (%)	*P* value	OR	95% CI LL	95% CI UL
ET-1	Male	GG	77 (78.6)	9 (8.5)	<0.001	Ref		
		GT	14 (14.3)	31 (29.2)	<0.001	23.178	5.618	95.629
		TT	7 (7.1)	66 (62.3)	<0.001	71.696	14.69	349.912
	Female	GG	71 (72.4)	7 (9.9)	<0.001	Ref		
		GT	17 (17.3)	15 (21.1)	0.185	2.764	0.614	12.433
		TT	10 (10.2)	49 (69)	<0.001	13.683	3.339	56.061

ECE	Male	CC	5 (5.1)	8 (7.5)	0.736	0.83	0.078	8.847
		CT	9 (9.2)	24 (22.6)	0.837	1.306	0.103	16.574
		TT	84 (85.7)	74 (69.8)	0.877	Ref		
	Female	CC	7 (7.1)	11 (15.5)	0.34	0.348	0.05	2.404
		CT	7 (7.1)	17 (23.9)	0.799	0.751	0.083	6.809
		TT	84 (85.7)	43 (60.6)	0.284	Ref		

Notes: CI = confidence interval; the analysis was adjusted based on age and BMI as covariates.

## Data Availability

There are no data available separately.
